# Scalable and DiI-compatible optical clearance of the mammalian brain

**DOI:** 10.3389/fnana.2015.00019

**Published:** 2015-02-24

**Authors:** Bing Hou, Dan Zhang, Shan Zhao, Mengping Wei, Zaifu Yang, Shaoxia Wang, Jiarui Wang, Xin Zhang, Bing Liu, Lingzhong Fan, Yang Li, Zilong Qiu, Chen Zhang, Tianzi Jiang

**Affiliations:** ^1^Brainnetome Center, Institute of Automation, Chinese Academy of SciencesBeijing, China; ^2^National Laboratory of Pattern Recognition, Institute of Automation, Chinese Academy of SciencesBeijing, China; ^3^Beijing Institute of Radiation MedicineBeijing, China; ^4^State Key Laboratory of Biomembrane and Membrane Biotechnology, and PKU-IDG/McGovern Institute for Brain Research, School of Life Sciences, Peking UniversityBeijing, China; ^5^Institute of Neuroscience, Shanghai Institutes for Biological SciencesShanghai, China; ^6^Queensland Brain Institute, The University of QueenslandBrisbane, QLD, Australia; ^7^Key Laboratory for NeuroInformation of Ministry of Education, School of Life Science and Technology, University of Electronic Science and Technology of ChinaChengdu, China; ^8^CAS Center for Excellence in Brain Science, Institute of Automation, Chinese Academy of SciencesBeijing, China

**Keywords:** optical tissue clearing, SeeDB, Urea, tract tracing, whole brain imaging, CLARITY, CUBIC, 3DISCO

## Abstract

Efficient optical clearance is fundamental for whole brain imaging. In particular, clearance of the brain without membrane damage is required for the imaging of lipophilic tracer-labeled neural tracts. Relying on an ascending gradient of fructose solutions, SeeDB can achieve sufficient transparency of the mouse brain while ensuring that the plasma membrane remains intact. However, it is challenging to extend this method to larger mammalian brains due to the extremely high viscosity of the saturated fructose solution. Here we report a SeeDB-derived optical clearing method, termed FRUIT, which utilizes a cocktail of fructose and urea. As demonstrated in the adult mouse brain, combination of these two highly water-soluble clearing agents exerts a synergistic effect on clearance. More importantly, the final FRUIT solution has low viscosity so as to produce transparency of the whole adult rabbit brain via arterial perfusion, which is impossible to achieve with a saturated fructose solution. In addition to good compatibility with enhanced yellow fluorescent protein, the cocktail also preserves the fluorescence of the lipophilic tracer DiI. This work provides a volume-independent optical clearing method which retains the advantages of SeeDB, particularly compatibility with lipophilic tracers.

## Introduction

Comprehensive depiction of structural layout and neuronal connectivity is indispensible for understanding how the mammalian brain functions. Due to high-throughput mechanical sectioning platforms and powerful automated three-dimensional imaging technologies, mesoscale atlases or connectomes of the entire mouse brain have been successfully reconstructed (Li et al., 2010; Oh et al., 2014). Despite these remarkable advances, the system-level relationship between brain structure and function often needs to be investigated, from a global perspective, in a whole brain rather than by reconstruction across preparations, in order to avoid loss of the detailed structure between sections (DeFelipe, 2010). However, sample opacity seriously limits the depth of imaging through the whole brain, particularly in adult animals. Recently, tissue optical clearing methods have opened a new avenue to extract cellular resolution information from unsectioned mammalian brains (Dodt et al., 2007; Hama et al., 2011; Becker et al., 2012; Erturk et al., 2012a; Chung et al., 2013; Ke et al., 2013; Kim et al., 2013; Kuwajima et al., 2013; Yushchenko and Schultz, 2013; Renier et al., 2014; Susaki et al., 2014; Tomer et al., 2014; Yang et al., 2014; Zhang et al., 2014).

Because tissue opacity results mainly from the scattering of light which occurs when the refractive index (RI) of the scatter differs from that of the medium (Helmchen and Denk, 2005), optical clearing methods usually yield tissue transparency via RI matching. A simple strategy is to merely utilize a clearing agent with a high RI approximating to that of fixed tissue (~1.5) (Dodt et al., 2007; Becker et al., 2012; Erturk et al., 2012a,b; Ke et al., 2013; Susaki et al., 2014). The alternative is to intentionally remove hydrophobic lipids which are the main source of light scattering in the fixed brain (Chung et al., 2013; Susaki et al., 2014). Among these techniques, fructose-based SeeDB does not disrupt the plasma membrane, and can therefore clear brain samples labeled with lipophilic dyes that are indispensible for neural tract tracing of post-fixed brains (Ke et al., 2013). However, the viscosity of saturated fructose (e.g., 130% wt/vol at 37°C) is extremely high, which limits its ability to permeate into brain samples as well as making solution preparation and manipulation difficult. Although incubation at a higher temperature could improve these permeability and fluidity issues, this would cause partial quenching of fluorescent proteins (Ke et al., 2013). Arterial perfusion-assisted delivery is also efficient for rapid diffusion of clearing agents across the whole brain (Yang et al., 2014), but extremely high viscosity prohibits application of SeeDB via arterial perfusion. In fact, it is difficult for SeeDB to clear larger mammalian brains, although SeeDB does render the adult mouse brain sufficiently transparent through immersion. Nevertheless, in mammals with larger brains, particularly in primates, investigation of the wiring diagram of the brain still relies heavily on tract tracing technologies. Thus, there is a compelling need to develop a clearing method which retains the advantages of SeeDB but overcomes its limitations.

Here we report an optical clearing method that uses a gradient of cocktail solutions, termed FRUIT, which is mainly composed of fructose and urea in variable proportions. FRUIT achieved greater tissue transparency and required a shorter clearing time than either fructose or urea alone. Urea-mediated tissue expansion could be controlled by increasing the concentration of fructose in the initial cocktail solution. Importantly, the presence of urea reduced the need for fructose. As compared with the highest concentration of SeeDB, the dynamic viscosity of the final FRUIT cocktail was lowered by over 97% so as to render the whole adult rabbit brain transparent via arterial perfusion. Under two-photon microscopy, the fluorescence of both enhanced yellow fluorescent protein (eYFP) and DiI was well preserved in the FRUIT-processed brain. The present study provides a new cocktail recipe which underlies a volume-independent and lipophilic tracer-compatible optical tissue clearing method.

## Materials and methods

### Makeup of the cocktail

To develop a clearing method which overcomes the limitations of SeeDB while retaining its advantages, we sought to make a cocktail comprising fructose and other available clearing agents which had been subject to careful verification and demonstrated satisfactory performance using adult mouse brain samples. The candidates were determined based on two principles. First, they needed to be compatible with the fluorescent proteins and tracers commonly used in neuroscience research. For whole-brain imaging, neurons of interest and their processes are usually recognized through the use of fluorescent labels. Thus, organic solvents were excluded due to concern regarding fluorescence quenching (Dodt et al., 2007; Becker et al., 2012; Erturk et al., 2012a,b). Given that high concentrations of fructose solutions are themselves relatively viscous, which makes it difficult to remove bubbles, the second principle required that the candidates were highly water-soluble but less bubble-generative. Accordingly, neither ionic nor non-ionic detergents were taken into consideration because they gave rise to bubbles during solution preparation and manipulation (Chung et al., 2013; Susaki et al., 2014). Based on the above two principles, urea was chosen as the other main ingredient of the cocktail solution, which was termed FRUIT.

**Figure 1:**
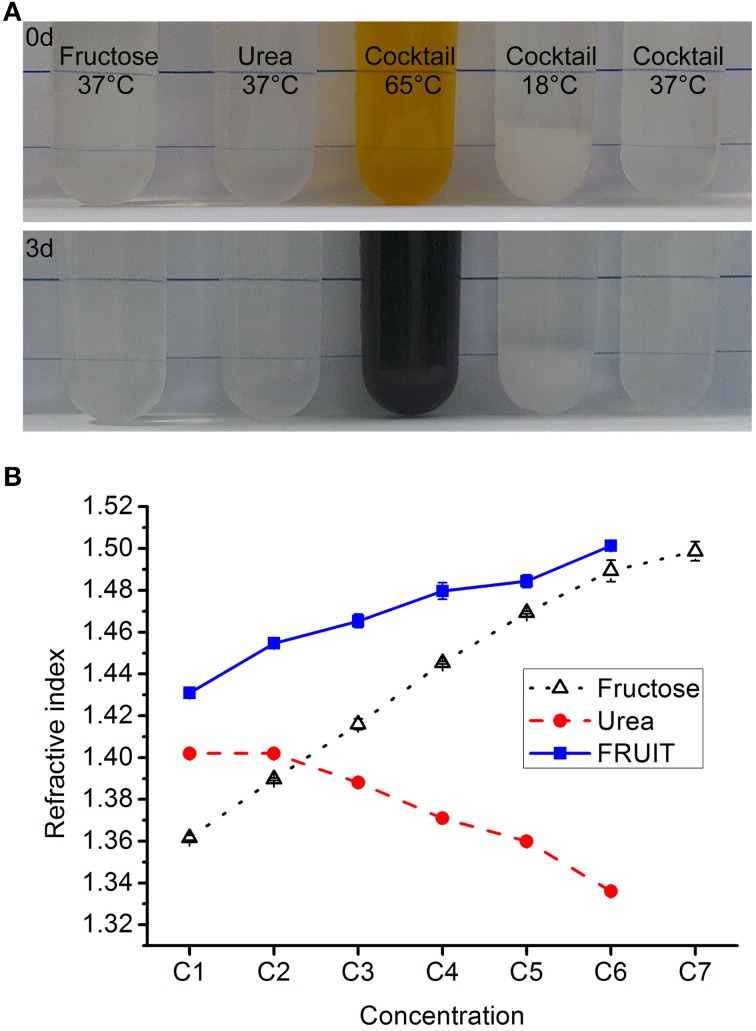
**Composition of the cocktail. (A)** The fructose solution (80% in wt/vol), urea solution (24% in wt/vol, equal to 4 M), and cocktail solution containing 80% fructose and 24% urea all remained stable in terms of appearance over 3 days at 37°C. In contrast, the cocktail solution turned from light brown to dark brown if kept at 65°C, whereas 8 g fructose and 2.4 g urea could not be fully dissolved in 10 ml of aqueous solution at 18°C. **(B)** The refractive index (RI) of the different clearing solutions presented as mean ± SD (*n* = 3). The composition of these solutions is shown in Supplementary Tables 1–3.

To determine whether or not fructose and urea can coexist, we conducted pilot tests to examine the simultaneous dissolution of fructose (80% wt/vol) and urea (24% wt/vol, equal to 4 M) in water. D-fructose, urea and other reagents were purchased from Aladdin Industrial Corporation (Shanghai, China) or Sinopharm Chemical Reagent Company (Beijing, China). Fructose was first completely dissolved in deionized water at about 65°C. After cooling to 37°C, urea was added to a final concentration as required. Notably, the mixture remained stable over 3 days at 37°C but not at 65°C, in terms of both appearance (Figure 1A) and RI. Given that SeeDB demonstrated general superiority over Sca*l*e (Ke et al., 2013), we decided to dissolve as much urea as possible into the ascending gradient of fructose solutions used in SeeDB so as to maximize the clearing action of the fructose. For convenience, the concentrations of the FRUIT solutions were determined in terms of their fructose concentration. It was found that fructose solutions to 83% (wt/vol) were capable of dissolving at least 4 M urea whereas a saturated fructose solution (130% wt/vol) could dissolve virtually no urea (Supplementary Figure 1). We then prepared a gradient of FRUIT solutions containing fructose and urea in variable proportions (Supplementary Table 1). For the purpose of comparison, the gradient of FRUIT solutions was initially designed following the gradient of fructose concentrations used in SeeDB. The urea was maintained at 48% (wt/vol, equal to 8 M) if possible or otherwise saturated, as the transitory use of 8 M urea significantly accelerated clearing of the brain without damage to fluorescent proteins (Hama et al., 2011). All FRUIT solutions contained 0.5% (wt/vol) α-thioglycerol. FRUIT solutions at a concentration of 100% (wt/vol) could be stored at 4°C without any crystallization. To control tissue expansion, 20~80% (wt/vol) FRUIT solutions were also prepared with different concentrations of PBS instead of water as indicated in Supplementary Table 1. The RIs of the solutions were measured at 18°C using an Abbe refractometer (INESA Instrument, Shanghai, China).

### Measurement of solution transmittance

**Figure 2:**
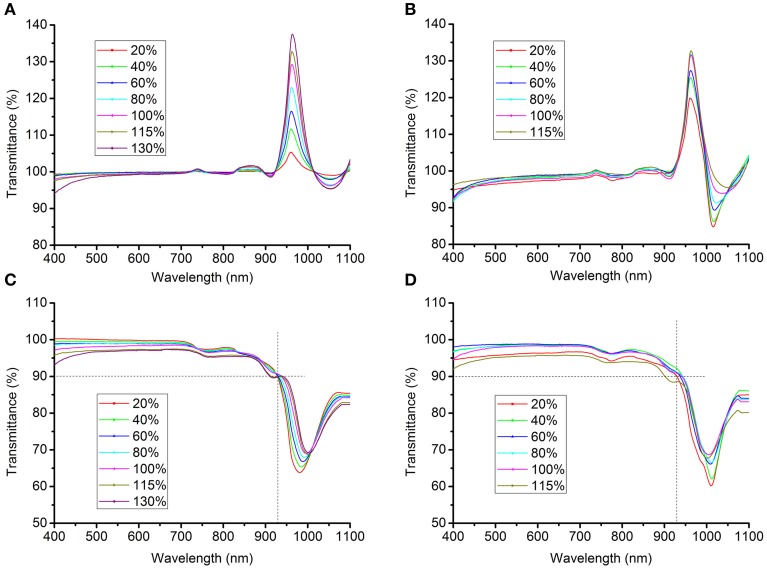
**Transmittance curves of clearing solutions. (A,B)** The transmittance of SeeDB **(A)** or FRUIT **(B)** solutions at different concentrations, normalized against pure water. The transmittance of the solutions exceeded the baseline in the band from 930 to 1000 nm, particularly at about 970 nm, because water itself substantially absorbs light at near-infrared wavelengths (Hale and Querry, 1973). **(C,D)** The corrected transmittance curves of the SeeDB **(C)** and FRUIT **(D)** solutions at different concentrations, normalized against air and the blank (see Materials and Methods).

The light transmittance of the clearing agents was measured using a spectrophotometer (UV2400, Sunny Optical Technology Group, Shanghai, China). The transmittance of solutions containing clearing agents was initially measured using pure water as the control. However, the transmittance of the solutions significantly exceeded baseline in the band from 930 nm to 1000 nm (Figures 2A,B), particularly at about 970 nm, because water itself substantially absorbs light at near-infrared wavelengths (Hale and Querry, 1973). This problem had probably been overlooked previously, as the endpoint of the transmittance curve was set before 950 nm in earlier studies (see Figure 1A in Hama et al., 2011 and Figure1D in Ke et al., 2013). To correct this bias, the transmittance of solutions was first normalized against air and the blank, respectively, after which the mean of the measured values was taken as the true transmittance.

### Animals

All animals were housed and treated in accordance with institutional guidelines. The experimental procedures and housing conditions were approved by the Animal Experiment Committee of the Beijing Institute of Radiation Medicine. C57BL/6N mice, Thy1-YFP (line H) mice in a C57BL/6N background and New Zealand rabbits were used for optical clearing.

### Optical clearing using FRUIT

**Figure 3:**
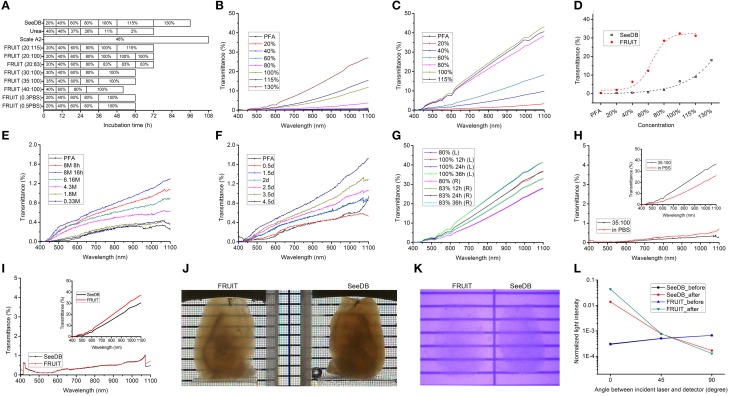
**Optical clearance of the adult mouse brain. (A)** Schematic diagram of different clearing procedures. FRUIT (20:115) denotes a FRUIT gradient starting at 20% and ending at 115% whereas FRUIT (0.5PBS) means that the FRUIT solutions from 20 to 80% were prepared with 0.5×PBS instead of water. **(B,C)** Transmittance curves of hemi-brains processed with SeeDB **(B)** and FRUIT (20:115) **(C)**, respectively. PFA, paraformaldehyde. **(D)** Comparison of normalized transmittance at 920 nm between hemi-brains treated with SeeDB and FRUIT (20:115) across different concentrations. **(E,F)** Transmittance curves of hemi-brains after treatment with a descending gradient of urea solutions **(E)** and Sca*l*e A2 solution **(F)**, respectively. **(G)** Transmittance curves of the left and right halves of a brain processed with FRUIT (20:100) and FRUIT (20:83), respectively. **(H)** Transmittance curves of the left and right halves of a brain before and after (upper right inset) treatment with FRUIT (35:100) or FRUIT solutions using 0.5 × PBS as the solvent (in PBS). The *y* axis in the inset is at ten times lower magnification than that in the main panel. **(I)** Transmittance curves of the left and right halves of a brain before and after (upper right inset) treatment with SeeDB or FRUIT (35:100). The *y* axis in the inset is at ten times lower magnification than that in the main panel. **(J,K)** Visible-light **(J)** and infrared **(K)** photographs of hemi-brains cleared with FRUIT (35:100) (left) or SeeDB (right). **(L)** Normalized laser intensity through the samples before and after treatment with SeeDB or FRUIT (35:100). The laser intensity from FRUIT-processed hemi-brains was lower than that through SeeDB-processed hemi-brains in the direction perpendicular to the incident laser, but was higher in the direction of the incident laser, implying that FRUIT was more effective at reducing scattering. The assessment of light scattering is schematically illustrated in Supplementary Figure 2.

The different FRUIT protocols used on the adult mouse brain are depicted in Figure 3A. The initially designed FRUIT (20:115) procedure was performed as follows. Adult mice over 70 days of age were deeply anesthetized with an intraperitoneal overdose of sodium pentobarbital (70 mg/kg body weight) and transcardially perfused with 1 × PBS followed by 4% (wt/vol) paraformaldehyde (PFA) in 1 × PBS. The whole brains were excised and then post-fixed in the same fixative at 4°C overnight. The brain samples were serially incubated in 20–30 ml of 20, 40, and 60% (wt/vol) FRUIT, each for 8 h in 50-ml conical tubes with gentle rotation (~4 rpm) at 37°C. The samples were then incubated in 80% (wt/vol) FRUIT for 12 h, 100% FRUIT for 12 h and finally 115% FRUIT for 24 h with gentle rotation at 37°C. In the case of the Thy1-YFP (line H) mice, the samples were covered with foil and protected from light during clearing. After noting that the samples showed volume expansion under the above conditions, we considered choosing an appropriate start concentration and elevating the ionic osmotic pressure in the FRUIT solutions to control FRUIT-mediated tissue expansion. In order to achieve maximal transparency without deformation, the optimal FRUIT protocol was established as follows: 8 h each in 35, 40, and 60% (wt/vol) FRUIT, 12 h in 80% (wt/vol) FRUIT and 24 h in 100% (wt/vol) FRUIT. Incubation at a temperature over 37°C is not recommended due to concern regarding the breakdown of FRUIT. Samples could be stored in 100% (wt/vol) FRUIT over 2 months at 4°C.

### Perfusion-assisted clearing using FRUIT

For perfusion-assisted clearance of the brain, 3 month old adult rabbits were anesthetized with an intravenous injection of sodium pentobarbital (35 mg/kg body weight) through the auricular vein. The rabbit was secured in a supine position and the common carotid arteries and internal jugular veins were exposed. The bilateral common carotid arteries were ligated and the internal jugular veins were opened. A needle was inserted into each common carotid artery at the distal segment to allow for cerebral perfusion of 1 × PBS followed by 4% (wt/vol) PFA in 1 × PBS. The rabbit brain was then sequentially perfused with 50 ml of 20, 40, 60, 80, and 100% (wt/vol) FRUIT solutions through an injection pump (ZT-500A1, Z&T Medical Treatment, Shenzhen, China) at a flow rate of 5~10 ml/h at room temperature. Arterial perfusion of SeeDB solutions could not be completed because 130% (wt/vol) fructose was too viscous to be infused even at 37°C.

### Optical clearing with seeDB

For clearing with SeeDB (Ke et al., 2013), PFA-fixed brain samples excised from the adult mouse were serially incubated in 20–30 ml of 20, 40, and 60% (wt/vol) fructose, each for 8 h with gentle rotation at 37°C. Samples were then incubated in 80% (wt/vol) fructose for 12 h, 100% fructose for 12 h, 115% fructose for 24 h and finally 130% fructose for 24 h with gentle rotation at 37°C. All fructose solutions contained 0.5% α-thioglycerol. Figure 3A schematically depicts the clearing procedure. For Thy1-YFP (line H) mice, the samples were covered with foil and protected from light during clearing.

### Optical clearing with scale and descending gradient urea solutions

Given that Sca*l*e has been systematically compared with SeeDB (Ke et al., 2013), we only compared the clearing competence between FRUIT and Sca*l*e A2 or a descending gradient of urea solutions within a similar incubation period. For clearing with Sca*l*e (Hama et al., 2011), PFA-fixed brain samples excised from the adult mouse were transferred to 20–30 ml of Sca*l*e A2 solutions containing 4 M urea, 10% (wt/vol) glycerol, and 0.1% (vol/vol) Triton X-100 and incubated at 37°C with gentle rotation for 4.5 days. For tissue clearance using a descending gradient of urea solutions, PFA-fixed brain samples excised from the adult mouse were serially incubated in 8 M (equal to 48% wt/vol) urea for 16 h, 6.16 M (37% wt/vol) urea for 8 h, 4.3 M (26% wt/vol) urea for 12 h, 1.8 M (11% wt/vol) urea for 12 h and 0.33 M (2% wt/vol) urea for 24 h with gentle rotation at 37°C. Figure 3A schematically illustrates these clearing procedures.

### Measurement of tissue transmittance

The light transmittance of the brain samples was measured using a spectrophotometer (UV2400, Sunny Optical Technology Group, Shanghai, China). As the light scattering of the adult brain is non-homogeneous due to the presence of highly myelinated structures (Ke et al., 2013), the medial-to-lateral transmittance of a hemi-brain was measured through its thickest part, with the midplane facing the light. Cleared hemi-brains without liquid can act as a convex lens due to the mismatch in RI between the air and the cleared samples (Susaki et al., 2014). To avoid such a possible convex lens effect, the samples were secured in quartz cuvettes filled with clearing agents with their midplane closely adherent to the sidewall of the cuvette, and their transmittance was normalized against the solutions containing different clearing agents. To ensure strict control, the brains were cut into equal left and right halves along the midline in several experiments, as illustrated in Figures 3G–I.

### Examination of sample deformation

For the measurement of sample deformation, the brains were cut into left and right halves along the midline, and the hemi-forebrains were excised. The samples were then incubated in SeeDB or FRUIT solutions as illustrated in Figure 3A. Photos of samples without liquid were taken before, during and after clearing, with their ventral plane on a glass plate. Based on top view photos, the size of the samples was examined with reference to the grid (5 × 5 mm) in the same picture.

### Assessment of light scattering

The brains were cut into equal left and right halves along the midline, and the samples were incubated in SeeDB or FRUIT (35:100). A laser beam at infrared wavelength (1064 nm was used due to the availability of a laser source) was positioned perpendicular to the midplane of the hemi-brains and the diameter of the laser spot on the midplane was adjusted to 2 mm. The laser intensity through the hemi-brains was measured in a dark room using a laser power meter (PD300, Ophir Photonics, Jerusalem, Israel) at angles of 0, 45 and 90 degrees from the incident laser, before and after clearing. The measured values were then normalized to the intensity of the incident laser for comparison. The assessment of light scattering is schematically illustrated in Supplementary Figure 2.

### Examination of solution viscosity

Dynamic viscosity of solutions was measured at room temperature (29°C) or 37°C using a viscometer (DV-S, Brookfield, Middleboro, MA). For the fluidity test, the pipettes were filled with 100% FRUIT or 130% (wt/vol) fructose solutions, and then maintained vertically to allow the solutions to fall by gravity at room temperature (18°C) or 37°C. The time for the solution with low viscosity to run out was recorded.

### DiI labeling

PFA-fixed brains were cut into left and right hemi-brains along the midline. As reported in the previous study (Lin et al., 2000), a small incision was made with a scalpel in the cingulate on each side, and a small DiI C_18_ (3) crystal (Invitrogen, Carlsbad, CA) was placed into the incision. The hemi-brain samples were then incubated in 2% (wt/vol) PFA in PBS at 37°C for 14 days, after which they were serially incubated in a gradient of SeeDB or FRUIT (35:100) solutions, respectively. During incubation and clearing, the samples were covered with foil and protected from light. Cleared samples were subjected to two-photon imaging using an upright microscope (FV10MP-BXD4CH, Olympus, Tokyo, Japan) and the images were acquired at 990 nm excitation.

### Two-photon imaging of cleared samples

The brain samples from Thy1-YFP (line H) mice were placed in a hand-made chamber filled with clearing agents. For SeeDB-treated samples, 130% (wt/vol) fructose was not used for immersion due to the fact that its extreme viscosity would cause an uneven RI distribution after evaporation of water during imaging and might impair image quality. Although previously suggested as an immersion agent (Ke et al., 2013), 2, 2′-thiodiethanol was also not used, due to lack of availability. As a result, 100% FRUIT (wt/vol, *RI* = 1.48) or 115% FRUIT (wt/vol, *RI* = 1.50) was used for immersion for FRUIT (35:100)- or SeeDB-cleared samples. An upright multiphoton microscope (FV10MP-BXD4CH, Olympus) was used for two-photon imaging with 920 nm excitation. Images at a resolution of 512 × 512 pixels were collected with a 25 × objective (Olympus, *NA* = 1.0, working distance = 4.0 mm). Image analysis and presentation were performed with FluoView 1000 software (Olympus).

## Results

### Synergistically improved clearing competence of the cocktail

According to the theory of RI matching, the proximity of the RI to that of fixed tissues (~1.5) predicts good clearing competence of agents (Ke et al., 2013). Our measurement revealed that the RIs of the FRUIT solutions were closer to 1.5 than those of the corresponding fructose or urea solutions (Figure 1B), suggesting that FRUIT may achieve better tissue clearance.

As tissue transparency was represented by light transmittance of brain samples under immersion in different aqueous solutions containing clearing agents, we first measured the transmittance of various clearing agents to assess their influence on the transmittance of samples. Unlike the RI, the transmittance did not differ significantly across solutions containing different clearing agents (Figures 2C,D), which suggests that light absorption by different solutions at most only slightly biases the transmittance of samples, albeit that this possibility could not be completely excluded.

We next subjected PFA-fixed adult mouse brain samples to different clearing procedures as indicated in Figure 3A. For the purpose of comparison, the gradient of FRUIT solutions was initially designed following the gradient of fructose concentrations used in SeeDB. The transmittance of hemi-brains treated with SeeDB and FRUIT (20:115) shared two common features. First, the transmittance gradually improved as the wavelength increased from 400 to 1100 nm (Figures 3B,C). Second, the transmittance also improved in a concentration-dependent manner, although at different rates (Figure 3D). Specifically, the transmittance of SeeDB-treated tissues was poor when the fructose concentration was lower than 80%, but improved sharply thereafter. This may explain why the typical SeeDB procedure ended with 130% fructose, despite the handling difficulty caused by extreme viscosity (Ke et al., 2013). In contrast, FRUIT-processed tissues already displayed considerable transmittance at the initial concentration (i.e., 20%) and continued to improve until 100%. These two features provide further assurance that the transmittance of cleared tissues reliably reflects actual tissue transparency.

We then compared the transparency of hemi-brains treated with different clearing agents. As predicted from the RIs, a gradient of FRUIT solutions achieved better tissue clearance than SeeDB (Figure 3D). However, both the Sca*l*e A2 solution and a descending gradient of urea solutions produced only negligible transmittance within a similar incubation period (Figures 3E, F), suggesting that the combination of fructose and urea exerted a synergistic rather than an additive effect on clearance competence.

We found that 115% FRUIT treatment did not provide any additional gain in tissue transparency (Figures 3C, D). To determine a suitable final concentration for the gradient, we then compared tissue transparency between 100% FRUIT and 83% FRUIT in which the concentration of urea was retained at 4 M (Supplementary Figure 1 and Supplementary Table 1). After 80% FRUIT, the former resulted in better tissue transparency (Figure 3G). Moreover, the best tissue clearance by the 100% FRUIT solution was achieved after 24 h and maintained thereafter (Figure 3G), suggesting that a 24 h incubation was sufficient for 100% FRUIT to achieve optimal clearance of PFA-fixed adult mouse hemi-brains.

### Controllable tissue deformation by the cocktail

**Figure 4:**
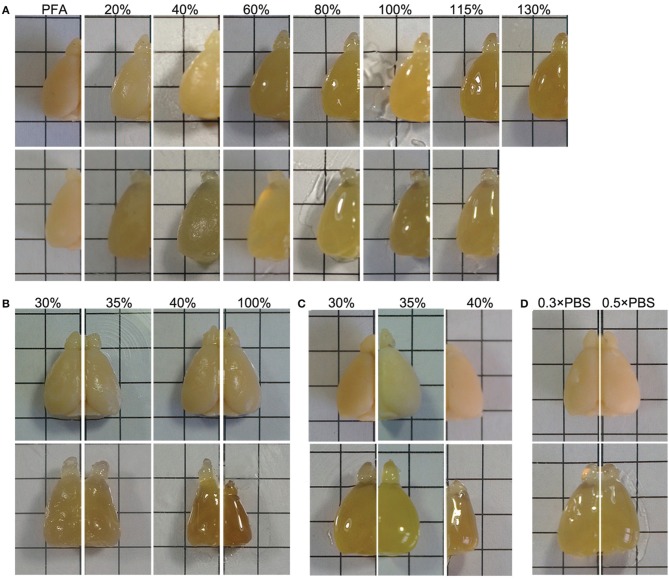
**Control of tissue deformation. (A)** The hemi-brains were sequentially treated with a gradient of SeeDB (upper row) or FRUIT (20:115) (lower row) solutions at 37°C. The final volumes of the brain samples show expansion after treatment with the FRUIT (20:115) but not the SeeDB protocol. For FRUIT-processed samples, the final size is seemingly equal to the size after 8 h incubation in the initial concentration of FRUIT, i.e., 20% FRUIT, regardless of the dynamic volume change across the gradient of FRUIT solutions. PFA, paraformaldehyde. **(B)** The hemi-brains before (upper row) and after (lower row) the 8 h incubation in single concentrations of FRUIT solutions at 37°C. Only 35% FRUIT is able to preserve the original size of the brain, whereas a lower or higher concentration causes expansion or shrinkage. **(C)** The hemi-brains before (upper row) and after (lower row) sequential treatment with the gradient of FRUIT solutions at 37°C starting from different concentrations. The gradient of FRUIT solutions starting from 35% preserved brain size well, whereas those with a lower or higher start concentration deformed the brain. **(D)** The hemi-brains before (upper row) and after (lower row) treatment with FRUIT solutions using 0.3 × PBS or 0.5 × PBS as the solvent at 37°C. 0.5 × PBS in 20–80% FRUIT solutions is sufficient to control expansion of the brain. This figure was set to demonstrate the changes of brain sizes in air in the epi-illumination mode (up-down). Brain transparency would be significantly improved by photographing the samples in solutions in the trans-illumination mode (down-up), as shown in Figures 3J, K. Grid size: 5 × 5 mm.

Tissue deformation commonly occurs during the process of optical clearing (Dodt et al., 2007; Hama et al., 2011; Erturk et al., 2012a; Chung et al., 2013; Ke et al., 2013). We noted that the final volume of brain samples also increased after treatment with FRUIT (20:115) (Figure 4A). Given that tissue expansion alone can reduce light scattering (Ke et al., 2013), there was a potential concern that the better tissue transparency afforded by FRUIT might be due to tissue expansion. We therefore, examined this possibility, and found that the brain stopped expanding at a FRUIT concentration of 40%, after which it partially recovered (Figure 4A). In contrast, the transparency of the same hemi-brain was continuously enhanced up to a concentration of 100% (Figures 3C, D). Therefore, it seemed unlikely that the better tissue transparency observed with FRUIT treatment was mainly mediated by tissue expansion. This concern was later completely resolved when FRUIT-caused tissue expansion was effectively controlled.

By comparing FRUIT with SeeDB and Sca*l*e, we concluded that FRUIT-mediated tissue expansion was mainly due to the high concentration of urea. Although reducing the level of urea in FRUIT solutions was beneficial for deformation control, this option was discarded because it would be detrimental to tissue transparency. As fructose can prevent tissue expansion through dehydration in a concentration-dependent manner (Ke et al., 2013; Susaki et al., 2014), one expansion-controlling strategy was to increase fructose in the starting concentration of the gradient of FRUIT solutions. We also found that the final size of the brain was seemingly equal to the size after 8 h incubation in the initial concentration of FRUIT, i.e., 20%, regardless of the dynamic volume change across the gradient of FRUIT solutions (Figure 4A). This finding inspired us to consider that choosing an appropriate starting concentration was enough to control FRUIT-mediated tissue expansion. We therefore, compared brain size after brief treatment in various concentrations of FRUIT solution. It was found that only 35% FRUIT was able to preserve the original size of the brain, whereas a lower or higher concentration caused expansion or shrinkage, respectively (Figure 4B). Notably, one-step treatment with 100% FRUIT caused severe shrinkage of the samples (Figure 4B). Based on these results, we modified the initial FRUIT concentration of the gradient. As expected, a gradient of FRUIT solutions starting from 35% preserved the brain size well, whereas those with a lower or higher start concentration deformed the brain (Figure 4C).

Brain deformation also reflects an imbalance of ionic osmotic pressure between the inside and outside of the brain. Thus, another expansion-controlling strategy we considered was to elevate the ionic osmotic pressure in the FRUIT solutions. Consequently, 20–80% FRUIT solutions were prepared with different concentrations of PBS instead of water (Supplementary Table 1). This revealed that 0.5 × PBS in 20–80% FRUIT solutions was sufficient to control for expansion of the brain (Figure 4D).

In order to achieve maximal transparency without deformation, we further compared brain transparency between the two simple but effective strategies for expansion control. After finding that the FRUIT procedure beginning at a concentration of 35% rendered the brain clearer than that using 0.5 × PBS as a solvent (Figure 3H), a gradient comprising 35, 40, 60, 80, and 100% FRUIT solutions (see Figure 3A), was finally selected as the optimal approach. However, it should be noted that the optimal procedure might vary depending on animal age, tissue volume, fixation time, solution temperature and other factors.

### Scalable clearance with eYFP and DiI compatibility

When tissue expansion was controlled, FRUIT (35:100) still achieved better brain transparency than SeeDB across the full spectrum from 400 nm to 1100 nm (Figure 3I), suggesting that FRUIT-mediated transparency occurred independent of brain expansion. Notably, the better tissue transparency afforded by FRUIT was more pronounced when examined under the infrared spectrum vs. visible light (Figures 3J, K), which facilitates two-photon imaging of the whole brain, particularly deep structures.

To explore a possible mechanism accounting for the greater transparency afforded by FRUIT, we assessed the light scattering of cleared brain samples, as the opacity of mammalian tissue results mainly from light scattering rather than light absorption (Ke et al., 2013). The laser intensity from FRUIT-processed hemi-brains was lower than that through SeeDB-processed hemi-brains in the direction perpendicular to the incident laser but higher in the direction of the incident laser (Figure 3L), meaning that FRUIT was more effective at reducing scattering.

**Figure 5:**
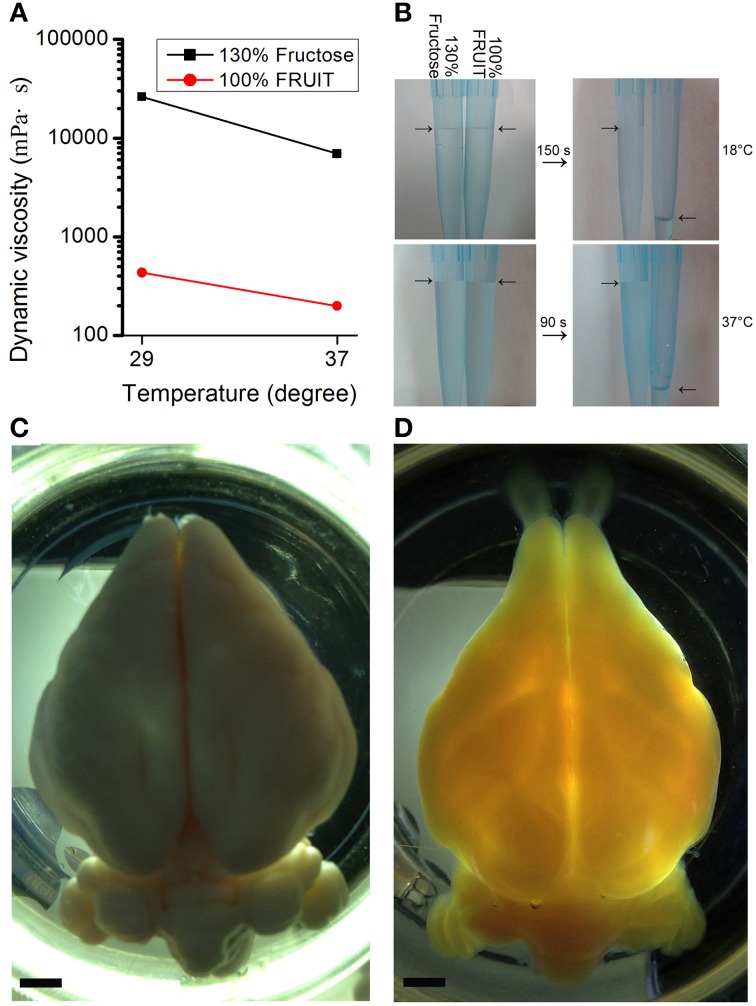
**Viscosity-dependent arterial perfusion of clearing agents. (A)** The dynamic viscosity of different clearing solutions presented as mean ± SD (*n* = 3). 100% FRUIT was 30 times less viscous than 130% fructose solution. **(B)** Fluidity test of the clearing solutions. 100% FRUIT ran out through the pipette within 150 s at 18°C (upper row) and within 90 s at 37°C (lower row) whereas 130% fructose hardly fell. **(C,D)** Adult rabbit brains after arterial perfusion of paraformaldehyde **(C)** or the gradient of FRUIT solutions **(D)**. Perfusion of FRUIT solutions rendered the whole brain transparent whereas 130% SeeDB was too viscous to be arterially perfused. An adult rabbit brain is about 20 times larger in volume than an adult mouse brain. Bar = 5 mm.

In addition to a synergistic action on tissue transparency, the combination of fructose and urea also resulted in other reciprocally beneficial consequences. Notably, the presence of urea reduced the amount of fructose required. Dynamic viscosity measurements revealed that whether at room temperature (i.e., 29°C) or under mild heating conditions (i.e., 37°C), 100% FRUIT was 30 times less viscous than a 130% fructose solution (Figure 5A). Low viscosity not only improved permeability but also accelerated clearance as evidenced by the fact that the total time required for FRUIT (35:100) to clear adult mouse brains was as short as 2.5 days, a reduction of approximately 40% as compared with SeeDB (Figure 3A). In addition, low viscosity also led to high fluidity (Figure 5B), which made it easy to prepare and exchange the FRUIT solutions as well as to handle samples immersed in them. More importantly, high fluidity made it possible to implement rapid diffusion of clearing agents across the whole brain via arterial perfusion. The gradient of FRUIT solutions, including the 100% concentration, could be smoothly infused into the common carotid artery so as to render the whole adult rabbit brain transparent (Figures 5C, D), whereas the highest concentration of SeeDB (130% fructose) was too viscous to be arterially perfused, suggesting that FRUIT could overcome the volume limitation on the brain imposed by SeeDB.

Given that urea itself leads to partial denaturation of proteins (Ke et al., 2013), a possible concern was that FRUIT might also cause damage to biological samples. However, it seemed that fructose rendered the brain resistant to denaturation by urea. Although Sca*l*e-treated samples are very fragile (Hama et al., 2011), FRUIT-treated samples did not display noticeable fragility.

**Figure 6:**
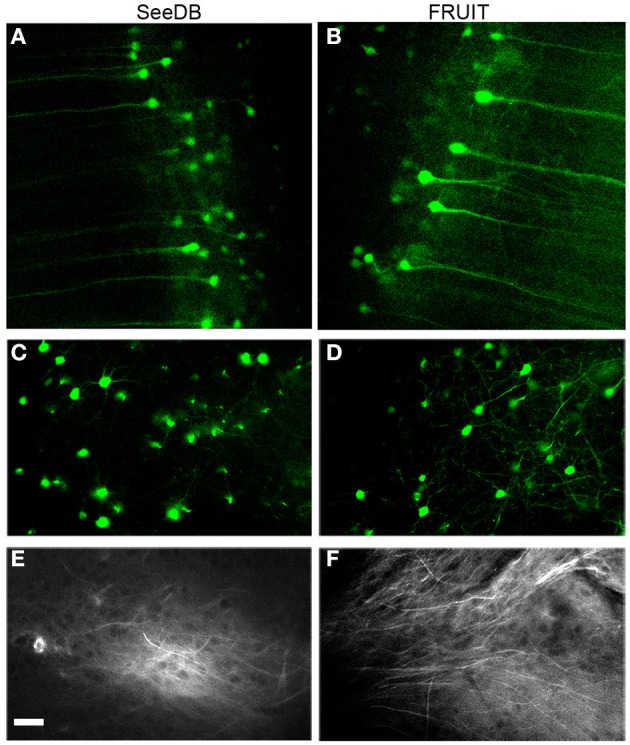
**Compatibility with fluorescent proteins or lipophilic tracers. (A, B)** Two-photon imaging of cortical pyramidal neurons in Thy1-eYFP (line H) mouse brains cleared with SeeDB **(A)** or FRUIT (20:115) **(B)**. The fluorescence of cytosolic eYFP was well preserved after treatment with FRUIT (20:115), but the neurons appeared to swell, which is in accordance with the gross appearance of the FRUIT (20:115)-treated brains (see Figure 4A). **(C,D)** Two-photon imaging of striatal neurons in Thy1-eYFP (line H) mouse brains cleared with SeeDB (C) or FRUIT (35:100), which effectively controlled the brain expansion **(D)**. **(E,F)** Two-photon imaging of DiI-labeled mouse brains after treatment with SeeDB **(E)** or FRUIT (35:100) which cause no deformation **(F)**. Like SeeDB, FRUIT preserved the fluorescence of fluorescent proteins or lipophilic tracers. Bar = 50 μm.

To determine whether or not FRUIT was compatible with the use of representative fluorescent proteins and lipophilic dyes, we examined FRUIT-cleared brains carrying these labels under two-photon microscopy. The fluorescence of cytosolic eYFP in the brains of Thy1-eYFP (line H) transgenic mice was well preserved after FRUIT treatment, similar to the result obtained after SeeDB treatment (Figures 6A–D). Given that eYFP has been reported to be the most susceptible fluorescent protein (Erturk et al., 2012a; Susaki et al., 2014), we speculated that FRUIT would also be valuable for the study of transgenic mice expressing other fluorescent proteins. Interestingly, and also similar to SeeDB (Ke et al., 2013), FRUIT could also clear brains labeled with the lipophilic dye DiI (Figures 6E,F), implying that the plasma membrane of FRUIT-processed brains remains intact and that FRUIT is suitable for tract tracing of post-fixed whole brains.

## Discussion

It was once considered impossible to extract cellular resolution information from unsectioned whole mammalian brains. Recently, tissue optical clearing methods have opened a new avenue to overcome this obstacle (Dodt et al., 2007; Hama et al., 2011; Becker et al., 2012; Erturk et al., 2012a; Chung et al., 2013; Ke et al., 2013; Kim et al., 2013; Kuwajima et al., 2013; Yushchenko and Schultz, 2013; Susaki et al., 2014; Tomer et al., 2014; Yang et al., 2014; Zhang et al., 2014). Previous clearing methods usually relied on a single clearing agent and rarely obtained a satisfactory balance between tissue transparency and other key parameters such as clearing time, deformation control, fluorescence preservation and operating simplicity (Kim et al., 2013; Yushchenko and Schultz, 2013). Theoretically, it is a promising strategy to make a cocktail of available clearing agents to achieve improved clearing performance without undesirable limitations. In fact, a cocktail strategy has been taken into consideration to direct the design of new clearing methods, such as ClearT (Kuwajima et al., 2013)and CUBIC (Susaki et al., 2014). Although a proposed cocktail does not necessarily translate into a better overall performance, our study provides a paradigm for evidence-based establishment of a new cocktail recipe that offers significantly improved outcomes over those obtained with the individual main ingredients alone. As the number of available clearing agents continues to grow, we believe that a cocktail strategy will prevail in order to ensure various desirable goals in whole-brain imaging studies.

According to predetermined principles regarding good fluorescence compatibility and effective bubble control, we chose urea as the other main ingredient for our FRUIT solutions. In theory, there are two different design strategies to create a cocktail of fructose and urea. One is to add increasing amounts of fructose into a constant concentration of urea solution, and the other is to dissolve as much urea as possible into an ascending gradient of fructose solutions. Given that SeeDB demonstrates general superiority over Sca*l*e (Ke et al., 2013), we adopted the latter strategy so as to maximize the clearing actions of fructose. Our choice is further justified by the following two points. First, keeping urea at a constant concentration (e.g., 4 M) would impose restrictions on the action of urea in FRUIT solutions at lower concentrations, as FRUIT to a concentration of 80% achieved considerable tissue transparency mainly through the use of urea at high concentrations above 4 M. Second, keeping urea constantly at 4 M would also reduce the solubility of fructose in FRUIT solutions at higher concentrations, whereas 100% FRUIT yielded greater brain transparency than 83% FRUIT.

Although the success of urea-based Sca*l*e is generally below that of fructose-based SeeDB (Ke et al., 2013), our study clearly demonstrates that urea plays a unique and important role in the applicability of FRUIT. Impressively, urea synergistically enhanced the clearing competence of fructose. We also noted two facts regarding tissue clearance by FRUIT. Firstly, to a concentration of 80%, FRUIT achieved much better tissue clearance than SeeDB. Secondly, treatment with 115% FRUIT (containing 0.33 M urea) following 100% FRUIT did not improve tissue transparency. These two facts suggest that urea may only exert an enhancing effect at certain concentrations. The generality and underlying mechanisms of this effect remain to be clarified.

The combination of fructose and urea also results in other reciprocally beneficial consequences. Among these, low viscosity is the most valuable achievement of FRUIT over SeeDB. Low viscosity improves permeability and accelerates clearance as evidenced by the reduced total time required for FRUIT, as compared to SeeDB, to clear adult mouse brains, in both cases under immersion conditions. Low viscosity also translates into high fluidity, which is a prerequisite for rapid diffusion of clearing agents across the whole brain via arterial perfusion. Remarkably, perfusion of a gradient of FRUIT solutions results in transparency of the whole adult rabbit brain which is about 20 times larger than its mouse counterpart and prohibitive for SeeDB. As perfusion-assisted release of clearing agents is not restricted by organ volume (Yang et al., 2014), we envision that FRUIT could even be applicable to the optical clearing of primate brains.

Although urea is reported to cause partial protein denaturation (Hama et al., 2011), our observations argue that this may not be true for urea-containing FRUIT. Firstly, unlike Scale-treated samples (Hama et al., 2011), FRUIT-treated samples were not noticeably fragile. Secondly, but more importantly, FRUIT was shown to be compatible with the lipophilic dye DiI, which implies that the plasma membrane of FRUIT-processed brains remains intact (Ke et al., 2013). These facts suggest that the fructose in the FRUIT solutions may be protective, helping to preserve the plasma membrane in the tissue. This feature distinguishes FRUIT as well as SeeDB from other optical clearing methods which are not suitable for tract tracing of post-fixed whole brains. However, SeeDB and our method would not facilitate immunostaining of the whole brain (Chung et al., 2013; Renier et al., 2014; Susaki et al., 2014; Tainaka et al., 2014; Yang et al., 2014), because they are not intentionally designed to remove lipids which render tissue poorly accessible to molecular probes, such as antibodies.

By means of arterial perfusion, FRUIT can achieve tissue clearance independent of brain volume limitations. Although CUBIC (Susaki et al., 2014) and a modified version of CLARITY (Yang et al., 2014) are also scalable clearing methods, both of them render the whole mammalian brain transparent by removing hydrophobic lipids, which makes it impossible to clear DiI- labeled brain samples. Thus, FRUIT represents a distinctive clearing method which is both independent of brain volume and compatible with lipophilic tracers. In the present study, we only conducted systemic comparisons between our method and SeeDB, in terms of clearing performance, deformation control, DiI compatibility and solution viscosity. Extensive comparisons across existing methods have been performed in previous literatures (see Table 1 in Kim et al., 2013, Table 1 in Yushchenko and Schultz, 2013, Supplementary Table 4 in Ke et al., 2013 and Table S1 in Yang et al., 2014). These comparisons suggest that no single clearing technique can satisfy the many goals of whole-brain imaging studies. Researchers may choose the most appropriate one out of the available clearing techniques or even adopt simultaneous or sequential use of them, depending on brain volume, labeling method, and observation tool.

It should also be kept in mind that the same clearing methods would perform differently under different experimental settings. Under confocal microscopy, SeeDB only marginally improved the imaging depth of cultured neurospheres that were 100 μm in diameter (Boutin and Hoffman-Kim, 2014), whereas the imaging depth of the adult mouse brain could reach almost 6000 μm after treatment with SeeDB under optimal two-photon microscopy (Ke et al., 2013). This example suggests that even the same clearing method may afford different imaging depths under different forms of microscopy.

Rapid development of whole-brain clearing methods also creates unprecedented demands for high-performance imaging platforms (Dodt et al., 2007; Hama et al., 2011; Becker et al., 2012; Erturk et al., 2012a,b; Chung et al., 2013; Ke et al., 2013; Kuwajima et al., 2013; Susaki et al., 2014; Yang et al., 2014). In order to achieve whole-brain imaging at a cellular resolution, it is necessary to utilize long working distance, high numerical aperture, and multi-immersion objectives (Yang et al., 2014). For samples as large as rabbit brains, it remains challenging to obtain whole-brain imaging using available two-photon microscopes. Although two-photon microscopy works on FRUIT-processed mouse brain samples, light-sheet illumination (Truong et al., 2011) could be integrated to significantly accelerate the image acquisition process.

### Conflict of interest statement

Bing Hou, Dan Zhang and Tianzi Jiang hold a patent (pending) for the FRUIT technique. The authors declare that the research was conducted in the absence of any commercial or financial relationships that could be construed as a potential conflict of interest.

## Supplementary material

Supplementary Figure 1 **Solubility of urea in a gradient of fructose solutions at 37°C**. Both fructose and urea are highly water soluble, and the solubility of urea decreases in converse with the fructose concentration. Fructose solutions to 83% (wt/vol) are able to dissolve at least 4 M urea, whereas saturated fructose solution can dissolve virtually no urea.

Supplementary Figure 2 **Schematic diagram of light scattering assessment**. A laser beam was positioned perpendicular to the midplane of the hemi-brains and the laser intensity through the sample was measured using a laser power meter placed at angles of 90 (D1), 45 (D2), and 0 degrees (D3) from the incident laser. M: the place of a monitor S: the sample.
