# Supplementary material for: Scalable and DiI-compatible optical clearance of the mammalian brain
Source: Front Neuroanat. 2015 Feb 24;9:19. doi: 10.3389/fnana.2015.00019 (PMC4338786; doi:10.3389/fnana.2015.00019)
Supplement: Supplementary file 1 [file Table1.DOCX]

Supplementary Table 1

Compositions of FRUIT solutions. The gradient of FRUIT solutions was initially designed following the gradient of fructose concentration used in SeeDB and composed of six concentrations, i.e., 20%, 40%, 60%, 80%, 100% and 115%. To control tissue expansion, 20~80% FRUIT solutions might also be prepared with different concentrations of PBS.

| No. | Ingredients (wt/vol) | | | Solvent |
| --- | --- | --- | --- | --- |
|  | Fructose | Urea | α-thioglycerol |  |
| C1 | 20% | 48% (8 M) | 0.5% | Deionized water |
| C2 | 40% | 48% (8 M)* | 0.5% | Deionized water |
| C3 | 60% | 37% (6.16 M) * | 0.5% | Deionized water |
| C4 | 80% | 26% (4.3 M) * | 0.5% | Deionized water |
| C5 | 100% | 11% (1.8 M) * | 0.5% | Deionized water |
| C6 | 115% | 2% (0.33 M) * | 0.5% | Deionized water |
| FU30 | 30% | 48% (8 M) | 0.5% | Deionized water |
| FU35 | 35% | 48% (8 M) | 0.5% | Deionized water |
| FU83 | 83% | 24% (4.0 M) * | 0.5% | Deionized water |
| FU3-20 | 20% | 48% (8 M) | 0.5% | 0.3×PBS |
| FU3-40 | 40% | 48% (8 M) * | 0.5% | 0.3×PBS |
| FU3-60 | 60% | 37% (6.16 M) * | 0.5% | 0.3×PBS |
| FU3-80 | 80% | 26% (4.3 M) * | 0.5% | 0.3×PBS |
| FU5-20 | 20% | 48% (8 M) | 0.5% | 0.5×PBS |
| FU5-40 | 40% | 48% (8 M) * | 0.5% | 0.5×PBS |
| FU5-60 | 60% | 37% (6.16 M) * | 0.5% | 0.5×PBS |
| FU5-80 | 80% | 26% (4.3 M) * | 0.5% | 0.5×PBS |

PBS: phosphate buffer solution. 1×PBS contained 137 mM NaCl, 8.1 mM Na_2_HPO_4_ and 1.9 mM KH_2_PO_4_.

*: The urea was saturated.
