# Supplementary material for: Scalable and DiI-compatible optical clearance of the mammalian brain
Source: Front Neuroanat. 2015 Feb 24;9:19. doi: 10.3389/fnana.2015.00019 (PMC4338786; doi:10.3389/fnana.2015.00019)
Supplement: Supplementary file 2 [file Table2.DOCX]

Supplementary Table 2

Compositions and refractive indices of an ascending gradient fructose solutions used in SeeDB.

| No. | Ingredients (wt/vol) | | Solvent |
| --- | --- | --- | --- |
|  | Fructose | α-thioglycerol |  |
| C1 | 20% | 0.5% | Deionized water |
| C2 | 40% | 0.5% | Deionized water |
| C3 | 60% | 0.5% | Deionized water |
| C4 | 80% | 0.5% | Deionized water |
| C5 | 100% | 0.5% | Deionized water |
| C6 | 115% | 0.5% | Deionized water |
| C7 | 130% | 0.5% | Deionized water |
