# Supplementary material for: Scalable and DiI-compatible optical clearance of the mammalian brain
Source: Front Neuroanat. 2015 Feb 24;9:19. doi: 10.3389/fnana.2015.00019 (PMC4338786; doi:10.3389/fnana.2015.00019)
Supplement: Supplementary file 3 [file Table3.DOCX]

Supplementary Table 3

Compositions and refractive indices of a descending gradient of urea solutions as used in FRUIT.

| No. | Ingredient (wt/vol) | Solvent |
| --- | --- | --- |
|  | Urea |  |
| C1 | 48% (8 M) | Deionized water |
| C2 | 48% (8 M) | Deionized water |
| C3 | 37% (6.16 M) | Deionized water |
| C4 | 26% (4.3 M) | Deionized water |
| C5 | 11% (1.8 M) | Deionized water |
| C6 | 2% (0.33 M) | Deionized water |
