# Supplementary figures and images for: Scalable and DiI-compatible optical clearance of the mammalian brain
Source: Front Neuroanat. 2015 Feb 24;9:19. doi: 10.3389/fnana.2015.00019 (PMC4338786; doi:10.3389/fnana.2015.00019)

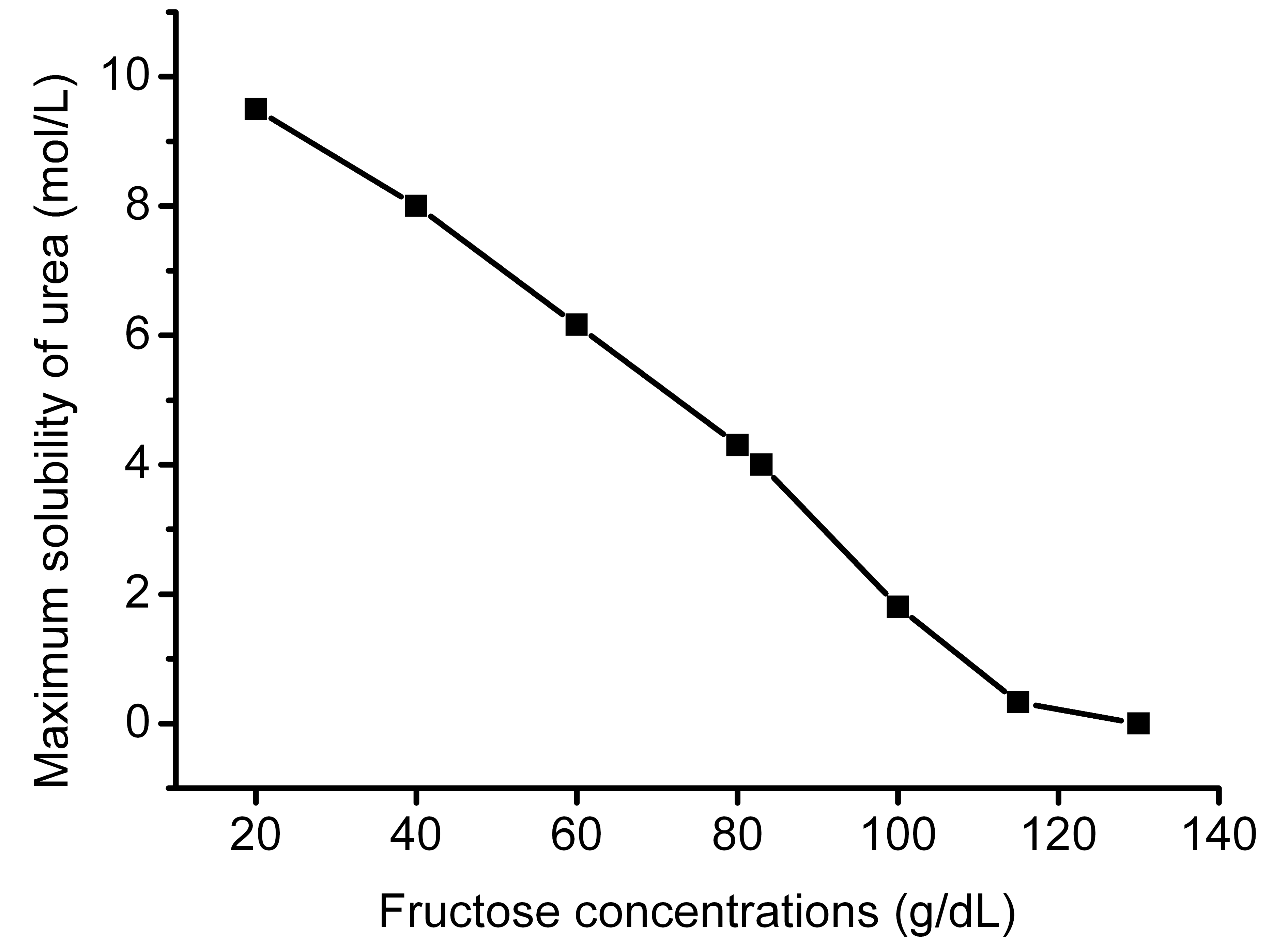

Supplement: Supplementary Figure 1 — Solubility of urea in a gradient of fructose solutions at 37°C. Both fructose and urea are highly water soluble, and the solubility of urea decreases in converse with the fructose concentration. Fructose solutions to 83% (wt/vol) are able to dissolve at least 4 M urea, whereas saturated fructose solution can dissolve virtually no urea. [file Image1.TIF]

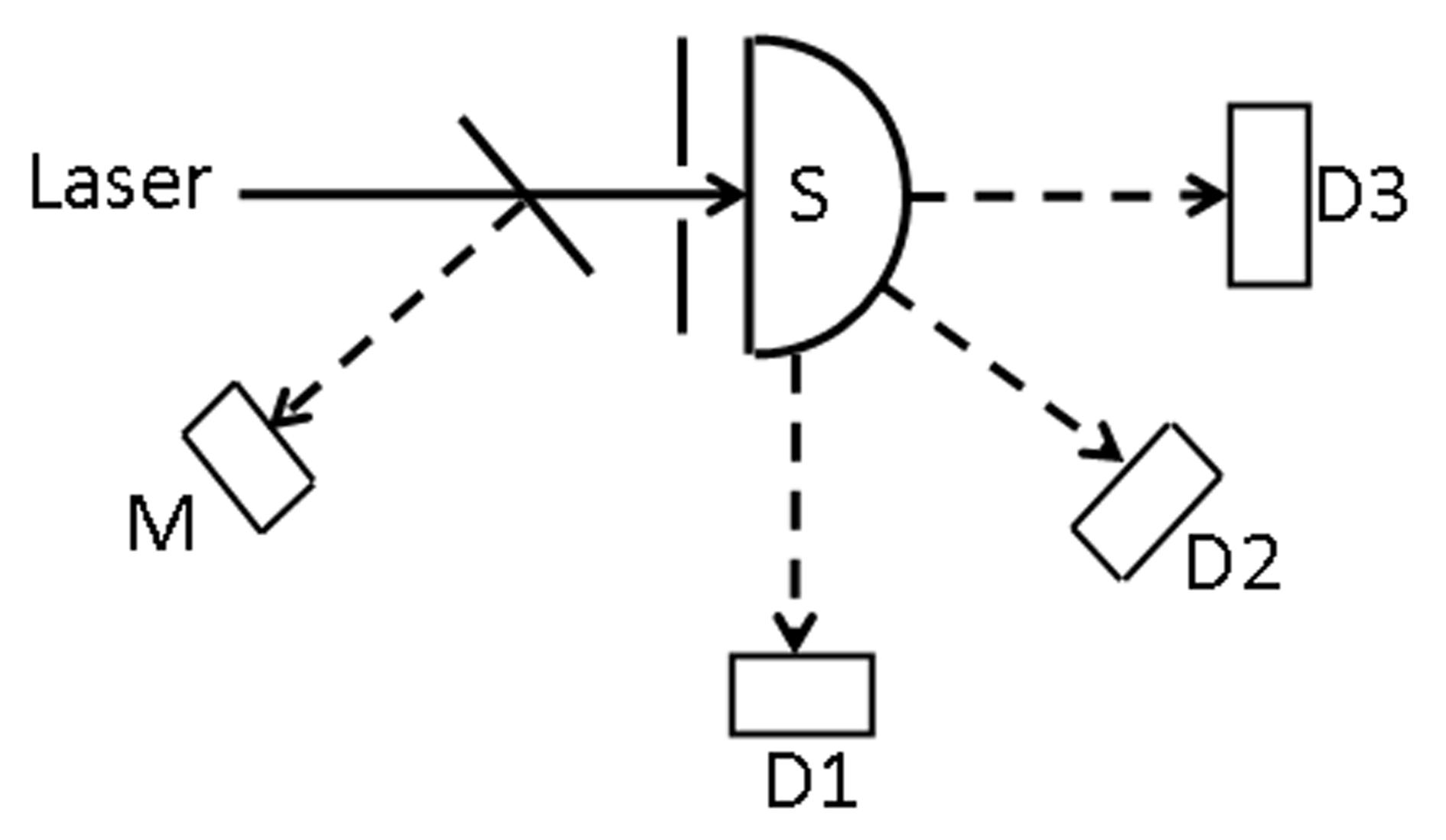

Supplement: Supplementary Figure 2 — Schematic diagram of light scattering assessment. A laser beam was positioned perpendicular to the midplane of the hemi-brains and the laser intensity through the sample was measured using a laser power meter placed at angles of 90 (D1), 45 (D2), and 0 degrees (D3) from the incident laser. M: the place of a monitor S: the sample. [file Image2.TIF]
